# Transcript Characteristics on the Susceptibility Difference of Bovine Respiratory Disease

**DOI:** 10.1155/2023/9934684

**Published:** 2023-05-04

**Authors:** Hang Cao, Chao Fang, Qiong Wang, Ling-Ling Liu, Wu-Jun Liu

**Affiliations:** College of Animal Science, Xinjiang Agricultural University, Urumqi, Xinjiang 830052, China

## Abstract

Bovine respiratory disease (BRD) is one of the major health issues in the cattle industry, resulting in significant financial crises globally. There is currently no good treatment, and cattle are made resistant to pneumonia through disease-resistant breeding. The serial blood samples from six Xinjiang brown (XJB) calves were collected for the RNA sequencing (RNA-seq). The obtained six samples were grouped into two groups, in each group as infected with BRD and healthy calves, respectively. In our study, the differential expression mRNAs were detected by using RNA-seq and constructed a protein–protein interaction (PPI) network related to the immunity in cattle. The key genes were identified by protein interaction network analysis, and the results from RNA-seq were verified using reverse transcription-quantitative polymerase chain reaction (RT-qPCR). A total of 488 differentially expressed (DE) mRNAs were identified. Importantly, the enrichment analysis of these identified DEGs classified them as mainly enriched in the regulation and immune response processes. The 16 hub genes were found to be related to immune pathways categorized by PPIs analysis. Results revealed that many hub genes were related to the immune response to respiratory disease. These results will provide the basis for a better understanding of the molecular mechanism of bovine resistance to BRD.

## 1. Introduction

Bovine respiratory disease (BRD) is one of the most serious health issues affecting the cattle sector globally, resulting in significant financial losses [1]. In the United States, the incidence of BRD is 70–80% with a mortality rate of 40–50% [2–5]. In China, the incidence of BRD is 50–100%, and the mortality rate is 20–50%. Extreme weather conditions, such as cold winters and temperature fluctuations between spring and autumn in Xinjiang China, contribute to increased susceptibility of calves to respiratory diseases including BRD [6]. The respiratory illness affects 40–80% of people, with a 60–90% fatality rate. In XinJiang, BRD is caused predominantly by infection with bacteria [6]. Unfortunately, the incidence of BRD and mortality rate increases annually despite the heavy use of antibiotics in the cattle industry [7, 8].

BRD is typically caused by bacteria and viruses. There are currently no effective vaccines, and the use of antibiotic treatment is also not ideal. Antibiotics can help prevent sickness; however, their overuse can result in the resistivity of these broad-spectrum antibiotics in human pathogens such as methicillin-resistant *Staphylococcus aureus* [9–11]. The selection of calves that are resistant to respiratory illness genetically may be an efficient method to minimize disease incidence [12]. Increased resistance may lead to reduction in the use of broad-spectrum antibiotics and the improved sustainability and appeal of resulting food products. Using transcriptome (RNA) sequencing, we sought to select cattle that were not susceptible to respiratory disease to reduce the prevalence and influence of disease in cattle.

RNA sequencing (RNA-seq) is a powerful tool that can be used to identify differentially expressed genes (DEGs) as potentially important indicators in breeding; analyze the category, structure, and expression changes in the transcription factors; and reveal the molecular regulatory mechanism of specific biological processes. Transcriptome sequencing has been widely used in disease research [13, 14]. In the present study, we analyzed blood samples obtained from calves with different level of resistance to BRD using RNA-Seq to identify key genes implicated in immune response. Our results provide reasonable foundation for elucidation of molecular mechanism relevant to resistance of Xinjiang brown cattle calves to respiratory disease.

## 2. Materials and Methods

### 2.1. Animal Assembly and Sample Retrieval

All experiments were performed in accordance with the guidelines established by the Animal Care Committee of Xinjiang Agricultural University (No. 2018017). Three diseased Xinjiang brown calves and three healthy Xinjiang brown calves were purchased from Altay, Xinjiang (Xinjiang, China). The calves (3 weeks old) were grown in equivalent environments, with ad libitum food and water, shared-housing arrangements. Blood samples were extracted from the jugular vein, flash-frozen in liquid nitrogen, and kept at −80°C. The susceptible and resistant groups were then housed separately.

In Xinjiang, China, the main factor that causes bovine pneumonia is *Pasteurella multocida* [6]. Animals were identified as BRD-positive based on clinical examination and serum haptoglobin concentration. Calves with at least one visual BRD sign, a rectal temperature ≥40°C, and abnormal lung sounds detected at auscultation were defined as BRD cases. Calves with no visual signs of BRD, a rectal temperature <40°C, no abnormal lung sounds detected at auscultation, and a serum haptoglobin concentration <0.25 g/L were classified as non-BRD animals for transcriptome analysis. At the same time, nasal swabs of healthy cattle and sick cattle were collected for isolation and identification of pathogenic bacteria. Through polymerase chain reaction (PCR) technology and sequencing, it was found that the selected positive sample was infected by the *P. multocida* type-A, and no bacteria were isolated from the healthy cattle. No drugs were used in the area/farm from which the animals were purchased for the present study.

### 2.2. RNA Isolation and Preparation

Initially, total RNA from whole blood was retrieved using trizol reagent (Invitrogen Co. Ltd, San Diego, USA) according to the reported kit procedure. However, the Agilent Bioanalyzer 2100 (Agilent Technologies, CA, USA) and the NanoPhotometer® spectrophotometer (IMPLEN, Westlake Village, CA, USA) were used to calculate the RNA purity in pricked samples. Consequently, after passing the quality test, RNase-free DNase set was further used for RNA purification, the RNA was purified using RNA purification kit (Tiangen, China). Additionally, the purified RNA quantification was assessed through Agilent Bioanalyzer Quality testing. For the following process, total RNA fulfilling the integrity criteria was selected, i.e., RINs >8.5 integrity and a 28S/18S ratio of >0.7.

### 2.3. Preparing Libraries, Assembling, and Sequencing Transcriptomes

According to the reported protocol, six RNA libraries for input data were generated using NEBNext® Ultra™ RNA Library Prep Kit for Illumina® (NEB, Ipswich, MA, USA). Poly-T oligo-attached to magnetic beads were used to extract mRNA from the prepared RNA library. In the NEBNext First Strand Synthesis Reaction Buffer, fragmentation was performed five times with divalent cations at a high temperature. M-MuLV Reverse Transcriptase and random hexamer primers were used to make first-strand cDNA (RNase H-). Following that, DNA polymerase I and RNase H were used to synthesize second-strand cDNA. The library fragments were purified using the AMPure XP technology to select cDNA fragments ranging from 150 to 200 bp (Beckman Coulter, Beverly, USA). Before PCR, 3 *μ*l of USER Enzyme (NEB, Ipswich, MA, USA) was used with size-selected, adaptor-ligated cDNA at 37°C for 15 minutes and then 5 minutes at 95°C. Phusion High-Fidelity DNA Polymerase, universal PCR primers, and index (X) primer were used in the PCR. According to the manufacturer's instructions, the prepared library quality was assessed using the Agilent Bioanalyzer 2100 system, and PCR products were purified (AMPure XP system). The TruSeq PE Cluster Kit v3-cBot-HS (Illumina) was used to cluster the index-coded samples on a cBot Cluster Generation System.

### 2.4. Data Analysis

Accordingly, the six cDNA libraries were sequenced through the Illumina NovaSeq 6000 sequencer. FastQC platform was utilized to evaluate the quality of obtained raw data. However, the original sequences filtered through FastQC were washed and screened by applying the selection criteria of low-quality data, N-containing reads, and linker sequences through Seqtk software. The filtered clean reads were compared to the *Bos taurus* reference genome using the Hisat 2.0 software (ARSUCD1.2). The expression level of genes was normalized to calculate fragments per kilobase of exon per million fragments mapped (FPKM) value using the program edge R. The resulting *P* values were adjusted using the Benjamini and Hochberg's approach for controlling the false discovery rate. The findings were screened differently and counted using the *P*-value <0.05, and log fold change |log_2_FC| > 1 was used as thresholds for significant differential expression in this study. The heat map of RNA expression was produced using the correlation of normalized gene-level FPKM values across samples with the heat map.

### 2.5. Functional Enrichment Analysis of DEGs

The Gene Ontology (GO) terms enrichment analysis for identified DEGs was performed through GOseq R [15]. The GO enrichment study was carried out by collecting all of the GO keywords significantly enriched, and further screening the DEGs based on their biological activities. All DEGs were mapped to GO words in the database (http://www.geneontology.org/), and gene numbers for each term were determined using the hypergeometric test to produce highly enriched GO terms. DEGs were considered significantly enriched by GO keywords with adjusted *P*-values less than 0.05. To perform pathway enrichment analysis and assess the statistical enrichment of DEGs in Kyoto Encyclopedia of Genes and Genomes (KEGG) (http://www.genome.jp/kegg/), the KO-Based Annotation System (KOBAS) program was used [15–18]. This approach was used to find genes involved in metabolic or signaling pathways that were highly overrepresented. The transcriptional factors (TFs) in the cattle genome were identified and classified using AnimalTFDB (http://www.bioguo.org/AnimalTFDB/).

### 2.6. Core Hub Protein–Protein Identification

The String database (http://string-db.org/) is a tool for determining how proteins interact. We used the String database to import the differential genes and evaluate the protein interaction relationship. The screening criteria were set at a confidence level of ≥0.4 and a degree of >1. Finally, the String analysis result was transferred into the Cytoscape module for network visualization.

### 2.7. Quantitative Reverse-Transcription PCR (RT-qPCR)

Real-time PCR was used to confirm the identified genes, with -actin serving as the internal control. Bioengineering Engineering (Shanghai) Co., Ltd. manufactured the primers, which were developed according to sequence specificity.  Table 1 encompasses the quantitative PCR primers used in the current study. The RNeasy Mini Kit (QIAGEN Gmbh, Hilden, Germany) was used to isolate total RNA from infected blood samples and infection-free controls at 3, 42, and 70 dpi. The RT2 First Strand Kit (QIAGEN Science, Maryland, USA) was used to reverse-transcribe DNase-digested total RNA (1 *μ*g) to single-strand cDNA, respectively. The qPCR reaction was performed on an ABI real-time PCR cycler (ABI 7500) using RT2 SYBR® Green ROX qPCR Mastermix (QIAGEN Gmbh, Hilden, Germany). For reverse transcription-quantitative polymerase chain reaction (RT-qPCR) verification, 16 genes were chosen at random.  Table 1 lists the forward (F) and reverse (R) primer pairs used to amplify the genes of interest in RT-qPCR procedures. The following conditions were used to carry out the amplification reactions: 95°C for 10 minutes, followed by 40 cycles of 95°C for 15 seconds, and 60°C for 1 minute. The following circumstances were used to conduct the melting curve analysis: to guarantee that a single product was amplified in each reaction, 1 minute at 95°C, 2 minutes at 65°C, and a gradual rise from 65°C to 95°C have been used. The relative gene expression level was evaluated by the 2^−△△Ct^ method.

## 3. Results

### 3.1. RNA Sequenced Data Analysis

We analyzed the blood samples of susceptible (S1, S2, S3) and resistant groups (R1, R2, R3) using RNA-seq. After stringent quality assessment (removal of connector and low-quality sequences) of raw RNA-seq data, we obtained 309,071,922 and 298,963,120 raw and clean reads, respectively, in six samples. The average percentage of clean reads was 95.98%. The contents of A and T were similar in the sequencing data, and the curves were coincident. The results of C and G were similar to those for A and T. Eventually, the obtained data were aligned against the reference genome. The assembly of these genomes showed the sequenced data were evenly distributed on the reference genome and accounted for 88.94–91.01% of the genome, with an average proportion of 90.41%. The above results indicated that the overall quality and randomness of the sequencing sequence were consistent and can be used for further downstream analysis.

### 3.2. Evaluation of DEGs

Significantly, a total annotated genes present in susceptible and resistant groups consisted of 14,247 and 13,956 genes, respectively. The maximum values of FPKM in two groups were 1.42 × 10^4^ and 0.93 × 10^4^, and the minimum values were 3.61 × 10^−3^ and 3.06 × 10^−3^, respectively. The above results indicate that the resistant group expressed fewer genes than the susceptible group, and the magnitude of gene expression was also lower (average and peak) in the resistant group.

**Figure 1:**
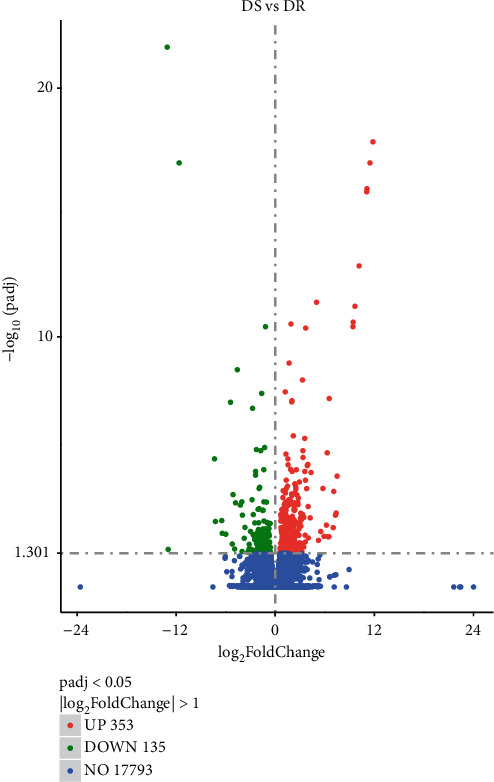
The volcano plot of differentially expressed genes (DEGs) between susceptible and resistant groups (UP: up-regulated; DOWN: down-regulated; NO: no significance).

**Figure 2:**
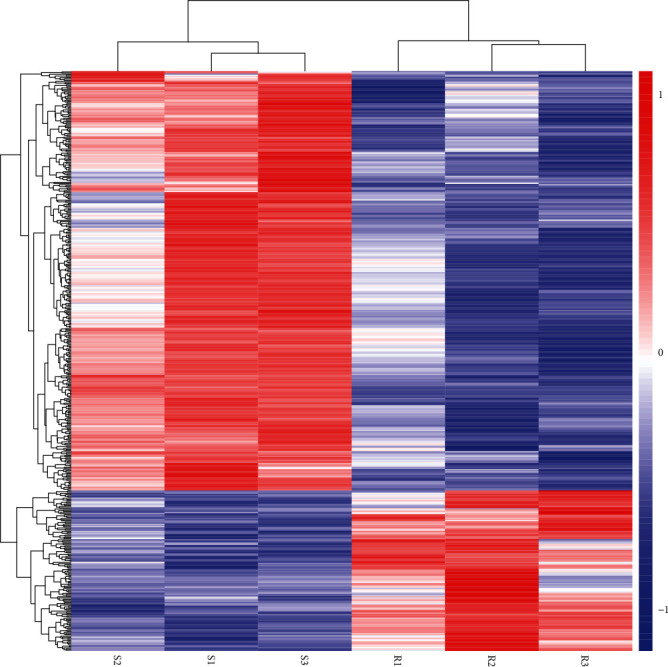
The hierarchical clustering (heat map) of differentially expressed genes (DEGs) between susceptible and resistant groups. The abscissa represents the sample, and the ordinate represents the different genes. Red indicates high gene expression, while blue indicates low gene expression. S1\S2\S3: susceptible groups. R1\R2\R3: resistant groups. Color scale bar represents the expression levels of the genes are shown as the log_2_ (FPKM + 1).

A total of 488 genes were found according to *P* < 0.05 and |log_2_FoldChange| > 1 when transcriptome data of two groups were compared. Subsequently, it was observed by RNA-seq data analysis that about 488 genes were differentially expressed (DE) in susceptible and resistant groups. From these, 353 (72.33%) DEGs showed upregulation while 135 (27.66%) DEGs were down-regulated, respectively. All obtained DEGs are highlighted in the volcano plot along with their hierarchical clustering (heat map) as shown in Figures 1 and 2, respectively.

### 3.3. Functional and Biological Processes Enrichment Analysis

**Figure 3:**
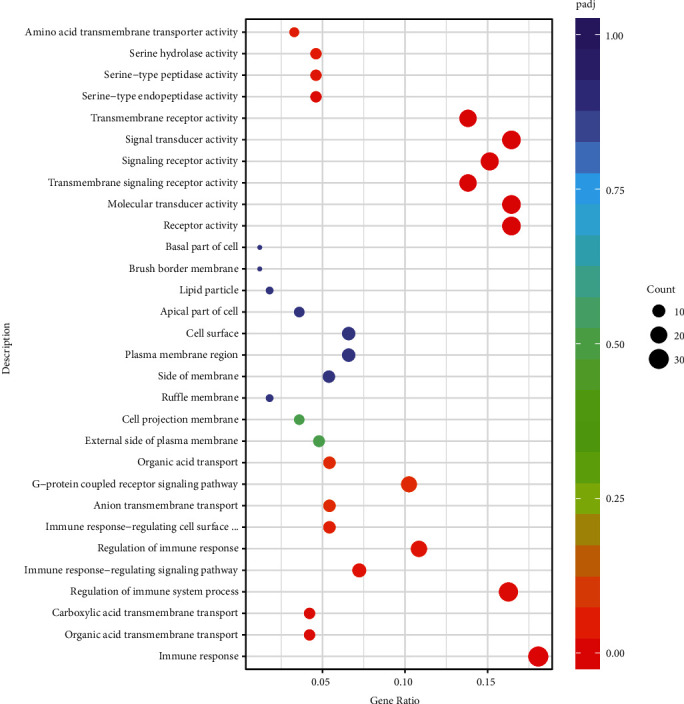
GO enrichment of differentially expressed genes (DEGs). Abscissa reflects gene ratios, while ordinates reflect GO pathway terms. Gene ratio: the ratio of the number of DEGs annotated on GO term to the total number of DEGs. Pajd: adjusted *p*-values.

Additionally, the enrichment analysis for identified DEGs was studied through GO terms, classifying these DEGs based on their cellular component (CC), biological process (BP), and molecular function (MF) GO terms. By using cut off value as *P* < 0.05, we recognized 68 genes significantly up-regulated in molecular transducer activity, receptor activity, serine-type endopeptidase, and peptidase activities, carboxylic acid transmembrane transport, organic acid transmembrane transport, and mainly in the immune response-regulating signaling pathway, regulation of immune response as shown in Figure 3 and Table 2.

Gene ratio: the ratio of the number of differential genes annotated to the GO number to the total number of differential genes. BP: biological process; MF: molecular function; CC: cellular component.

**Figure 4:**
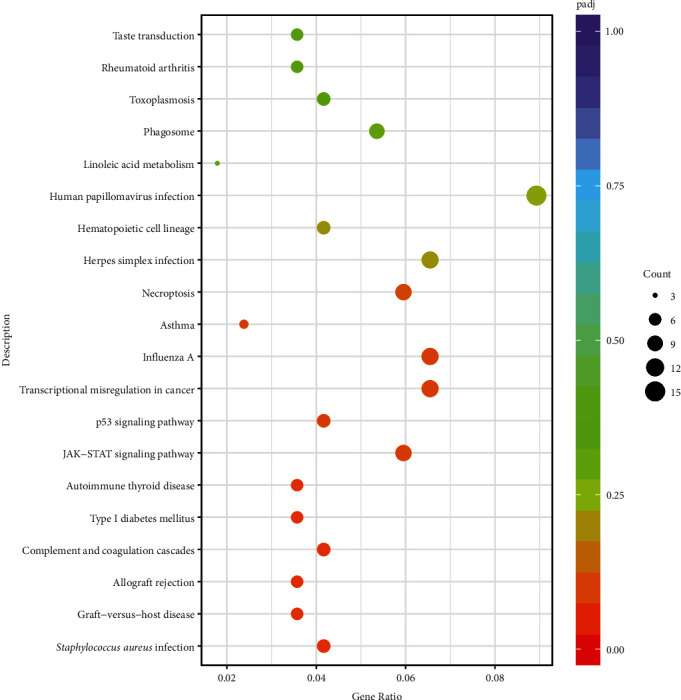
Signaling pathways enrichment of differentially expressed genes (DEGs). Abscissa reflects gene ratios, while ordinates reflect KEGG pathway. Gene ratio: the ratio of the number of DEGs annotated on KEGG pathway to the total number of DEGs. Pajd: adjusted *p*-values.

Furthermore, metabolic pathway analysis was performed for these identified DEGS utilizing the KEGG server. It showed that DEGs were significantly enriched with 52 numerous metabolic and signaling pathways, i.e., fructose and mannose metabolism, cytokine–cytokine receptor interaction pathway, nitrogen metabolism, including the IL-17 signaling pathway, nucleotide-binding oligomerization domain (NOD)-like receptor signaling pathway, influenza A, Tumor Necrosis Factor (TNF) signaling pathway, Nuclear factor-kappa B (NF-*κ*B) signaling pathway, Cyclic adenosine 3′,5′-monophosphate (cAMP) signaling pathway, and Janus kinase/signal transducers and activators of transcription (JAK/STAT) signaling pathway (Figure 4 and Table 3).

### 3.4. Gene Interactions Network Analysis

**Figure 5:**
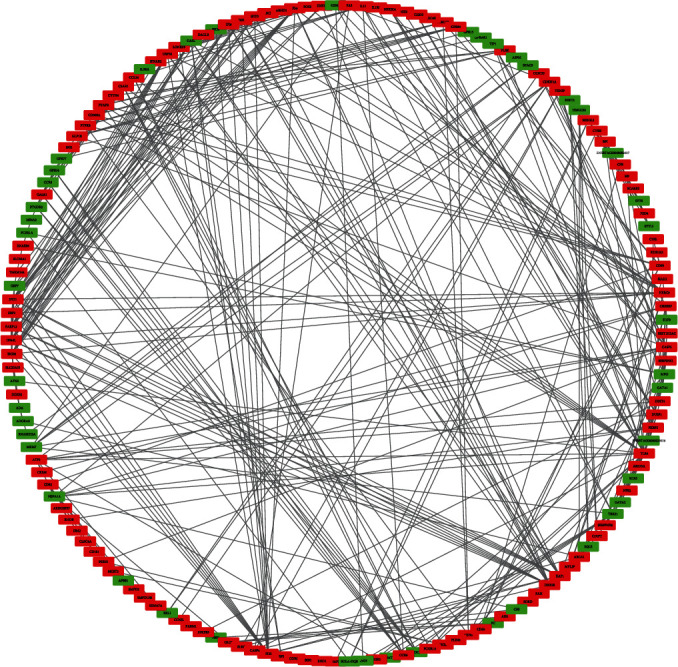
Protein–protein interaction networks (red: up-regulated; green: down-regulated).

STRING (http://string-db.org/) tools were used to predict protein interactions among the 488 DEGs. The circular arrangement in Cytoscape software (http://www.cytoscape.org/; version 3.8.0) was used to distribute all genes (Figure 5).

**Figure 6:**
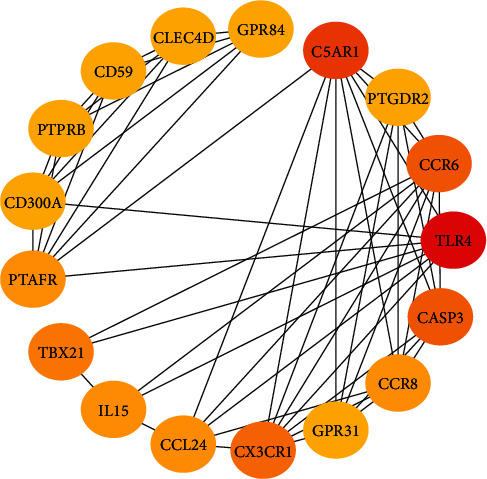
Interactions between the 17 hub proteins in the cluster with the highest score. Note: the scores of interactions between proteins rank by color order as follows: red, orange, and yellow.

Among these DEGs, the top 17 hub genes were selected based on their properties. The network and interaction analysis of these hub proteins were further generated and visualized through CytoHubba plugin of Cytoscape (Figure 6).

### 3.5. Quantitative Reverse-Transcription PCR (RT-qPCR) for Validation of RNA-Seq Data

**Figure 7:**
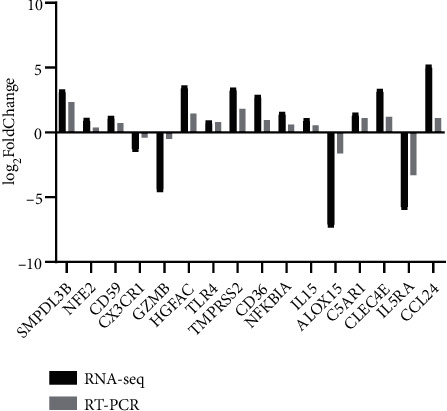
Validation of RNA-seq data by RT-qPCR.

Moreover, transcriptome sequenced data was further evaluated to check its accuracy. For this purpose, 16 DEGs consisting of 13 up-regulated (SMPDL3B, IL5RA, NFE2, CD59, CCL24, CLEC4E, TLR4, HGFAC, CD36, NFKBIA, TMPRSS2, IL15, and C5AR1) and 3 down-regulated genes (ALOX15, GZMB, and CX3CR) were randomly selected. The expression patterns of these 16 genes were comparable with transcriptome sequencing (Figure 7), demonstrating data accuracy.

## 4. Discussion

The survival ratio of calves has a direct bearing on overall economics of cattle production. Respiratory disease has the highest morbidity and fatality rate in calves [19]. Although there are a few studies [19, 20] on the molecular regulatory mechanism of BRD using omics techniques, however, studies on the calf model are still rare. The pathogenesis of BRD has a complex regulatory mechanism. And different pathogens may have various regulatory mechanisms. Our current understanding of its mechanistic basis remains limited. In the present study, we focused on the calf respiratory disease caused by *P. multocida*.

*Pasteurella* was previously found to be the main infectious pathogen among calves in the Xinjiang region of China [6]. It is one of the most common pathogenic bacteria associated with BRD potentially causing inflation related to platelet aggregation [21].

We found that most of the DEGs were up-regulated in calves with respiratory disease and were associated with innate immunity. The DEGs may be used to predict respiratory diseases caused by bacterial pathogens and as molecular markers for breeding. The identification of key upstream regulatory factors may provide possible targets for developing new molecular therapies [22]. Sixteen key candidate genes related to immune response and function were found by RNA-seq analysis of susceptible and resistant groups. Additionally, *TLR4*, *CD59*, *CD36*, *NFκBIA*, and *C5AR1* were revealed as the key regulator genes in BRD through protein–protein interaction network analysis.

Previous studies have shown the inflammatory response of endotoxin-induced bovine mastitis. Furthermore, it is reported that the NF-*κ*B signaling pathway activated by TFs is mainly involved in microbial infection in mice [23–29]. These pathways may be activated by the toll-like receptors (TLRs) mainly function in adaptive immunity through T-lymphocytes [30–35]. When bacteria invade the lungs of calves, biological signals induce the formation of heterodimers of TLR4 and TLR6, which rapidly trigger an inflammatory response [36]. It is believed that specific immune factors can be produced to protect against pathogens that invade the lungs by inducing the NF-*κ*B signaling pathway [30, 32]. In light of the foregoing information, it's worth noting that TLR4 was up-regulated in the current study, implying that TLR4's immunological mechanism was activated in the vulnerable group and may have led to an inflammatory response. In addition, *NFκBIA* was also identified in this study, which is an important regulatory factor in the NF-*κ*B signaling pathway [31].

*SMPDL3B*, was one of the 16 candidate genes that were up-regulated in the present study. It indicates that *SMPDL3B* may have inhibited the function of *TLR4* and blocked the immune response in the susceptible group [35]. This may have resulted in the immunosuppression of the susceptible group.

The identified marker genes were observed to be functionally enriched in immune regulation and serine activity classified through GO terms. Inflammation is closely related to immunity, and infection evokes the body's immune response, leading to the stimulation of the host cell immune signaling pathway, promoting the inflammatory response, and increasing the release of *IL-6* pro-inflammatory factors [32]. Previous studies have found that after infection with bacterial pneumonia, the concentrations of *IL-2* and *TNF-α* in susceptible types were increased significantly and gradually decreased as inflammation declined [32]. The levels of immune factors may be related to the severity of the disease [26, 33]. Increasing evidence has shown activation of immune response in human host by the expression of inflammatory cytokines [34].

KEGG pathway enrichment analysis revealed that the NF-*κ*B signaling pathways were significantly enriched. TLR4 was discovered to be a critical candidate gene that has been reported to activate NF-B through Lipopolysaccharide (LPS) and promote IL-1, IL-6, and IL-8 inflammatory factors overexpression [36]. These factors have a role in innate immune responses and disease resistance, as well as activating innate and adaptive immunological responses and aiding in pathogen protection [26, 34].

## 5. Conclusion

In the present study, we used RNA-seq to perform a comparative transcriptomic analysis of peripheral blood in Xinjiang Brown Cattle calves susceptible and resistant to BRD. We established the gene expression profile of Xinjiang brown cattle. A total of 488 DEGs were screened out. From these, mRNA expression of 16 key candidate genes related to immune response, i.e., *SMPDL3B*, *IL5RA*, *NFE2*, *CD59*, *CX3CR1*, *CLEC4E*, *TLR4*, *CD36*, *NFκBIA*, *C5AR1 GZMB*, *HGFAC*, *IL15*, *TMPRSS2*, *ALOX15*, and *CCL24* was validated using RT-qPCR. The RT-qPCR gene expression trend was consistent with the results of RNA-seq. According to core hub, genes were analyzed by degree methods, we infer that *TLR4*, *SMPDL3B*, and *NFκBIA* may be important regulatory genes in calves' immune response to respiratory disease. Our findings provide a stable platform for a deeper understanding of the molecular mechanism behind calf BRD resistance in Xinjiang brown cattle.

## Figures and Tables

**Table 1:** Gene primers for qPCR.

Number	Gene name	Gene sequence number	Primer sequence (5′ to 3′)	Amplified fragment length/bp	Annealing temperature/°C
1	*IL5RA*	ENSECAG00000016962	F-AGTGAGAGCAGCAGTGAG	150 bp	60
			R-ATTAACAAGATGAAGCAGATGG		
2	*NFE2*	ENSECAG00000019715	F-GACGCTGAATCTCTTGAG	163 bp	60
			R-AGGGTTCTAAGGCTAAGG		
3	*CD59*	ENSECAG00000001568	F-GTGTTGGAAGTACGAAGAATG	144 bp	60
			R-GTGAGCAGCAGCAGTATC		
4	*CX3CR1*	ENSECAG00000008165	F-CAGACACTCAATCTCTATGACTTC	195 bp	60
			R-ATGGCAACACAGGACAGC		
5	*CCL24*	ENSBTAG00000026275	F-GGTCCAGAAGTATGTAAAGAAC	98 bp	60
			R- CCAGGTGTCTCCAGAAGG		
6	*GZMB*	ENSBTAG00000039813	F- CCAGACTATAATGATGAGAC	180 bp	60
			R- TGTAGAGGGCATATTTACC		
7	*CLEC4E*	ENSBTAG00000012185	F-GGTCAGTGGCAATGGGTAG	108 bp	60
			R-CCTTATGGTGGCACAGTCC		
8	*TLR4*	ENSBTAG00000006240	F-CATCATCTTCATCGTCCTG	190 bp	60
			R-ATCTGCTGTTCCTTCTGG		
9	*HGFAC*	ENSBTAG00000017335	F-CAAGAGACACAAGAAGAGG	104 bp	60
			R-TGTTCCCGATGTAGATGG		
10	*CD36*	ENSBTAG00000017866	F-ATTGAAGCATTGAAGAATCTGAAG	167 bp	60
			R-ACCACACCAACACTGAGC		
11	*NFKBIA*	ENSBTAG00000016683	F-ACCTGGTGTCACTCCTATTG	169 bp	60
			R-TCCTCATCCTCGCTCTCC		
12	*TMPRSS2*	ENSBTAG00000009132	F-CCAGTGTGTCTACCCAATC	104 bp	60
			R-AGGTCATCTGAGGTCTTCC		
13	*IL15*	ENSBTAG00000018200	F-AGGCTGGCATTCATGTCTTC	165 bp	60
			R-AATTGGGATGAGCATCACTTTC		
14	*ALOX15*	ENSBTAG00000011990	F-CACTGCCGAACTTCCATC	150 bp	60
			R-CCTCCCTGAACTTCTTTAGC		
15	*C5AR1*	ENSBTAG00000020872	F-CGTGAATATGCGGAGAAG	89 bp	60
			R-CAGATTGTAAGCGTGACC		
16	*SMPDL3B*	ENSBTAG00000012997	F-CCAACAATCCAGGCATCC	86 bp	60
			R-GCTCAGGTTCAAGAAGTAGG		

**Table 2:** GO function analysis of DEGs.

GO	Categories	Gene ratio	*P*	Genes
Immune response	BP	30/166	1.85 × 10^−6^	SMPDL3B/CLEC4E/ALOX15/PIGR/TLR4/CCR8/IFNAR2/CX3CR1/MS4A2/IL5RA/TBX21/C5AR2/CCL24/FAS/BoLA/CASP4/CTSH/CD36/C5AR1/PRG3/CFB/CMTM3/NFKBIZ/ARID5A/ZBP1/ADA/RARA
Organic acid transmembrane transport	BP	7/166	1.98 × 10^−5^	SLC16A11/SLC38A1/IRS2/MGC157082
Carboxylic acid transmembrane transport	BP	7/166	1.98 × 10^−5^	SLC16A11/SLC38A1/IRS2/MGC157082
Regulation of immune system process	BP	27/166	3.14 × 10^−5^	SMPDL3B/CLEC4E/ALOX15/PIGR/TLR4/EVI2B/GATA2/TSC22D3/IRS2/MS4A2/TBX21/C5AR2/CCL24/HERC5/BoLA/CTSH/OCSTAMP/PIAS3/VEGFB/C5AR1/CFB/CMTM3/NFKBIZ/ZBP1/ADA/RARA
Immune response-regulating signaling pathway	BP	12/166	7.72 × 10^−5^	SMPDL3B/CLEC4E/PIGR/TLR4/MS4A2/C5AR2/CTSH/C5AR1/CMTM3/NFKBIZ/ADA
Regulation of immune response	BP	18/166	0.000104629	SMPDL3B/CLEC4E/ALOX15/PIGR/TLR4/MS4A2/TBX21/C5AR2/BoLA/CTSH/C5AR1/CFB/CMTM3/NFKBIZ/ZBP1/ADA/RARA
Receptor activity	MF	25/152	4.85 × 10^−6^	CLEC4E/GPR34/ADGRE3/PIGR/TLR4/CCR8/ANTXR2/TMPRSS2/PTAFR/IFNAR2/CX3CR1/FZD4/IL5RA/C5AR2/FAS/PLXNB2/GPBAR1/SCARB2/CTSH/GPR84/ABCA1/C5AR1/RARA
Molecular transducer activity	MF	25/152	4.85 × 10^−6^	/CLEC4E/GPR34/ADGRE3/PIGR/TLR4/CCR8/ANTXR2/TMPRSS2/PTAFR/IFNAR2/CX3CR1/FZD4/IL5RA/C5AR2/FAS/PLXNB2/GPBAR1/SCARB2/CTSH/GPR84/ABCA1/C5AR1/RARA
Transmembrane signaling receptor activity	MF	21/152	6.11 × 10^−6^	/GPR34/ADGRE3/PIGR/TLR4/CCR8/ANTXR2/PTAFR/IFNAR2/CX3CR1/FZD4/IL5RA/C5AR2/FAS/PLXNB2/GPBAR1/CTSH/GPR84/ABCA1/C5AR1
Signaling receptor activity	MF	23/152	8.93 × 10^−6^	CLEC4E/GPR34/ADGRE3/PIGR/TLR4/CCR8/ANTXR2/PTAFR/IFNAR2/CX3CR1/FZD4/IL5RA/C5AR2/FAS/PLXNB2/GPBAR1/CTSH/GPR84/ABCA1/C5AR1/RARA
Signal transducer activity	MF	25/152	1.02 × 10^−5^	CLEC4E/GPR34/ADGRE3/PIGR/TLR4/CCR8/ANTXR2/PTAFR/IFNAR2/CX3CR1/FZD4/IL5RA/C5AR2/CCL24/FAS/PLXNB2/CASP4/GPBAR1/CTSH/GPR84/ABCA1/C5AR1/RARA
Transmembrane receptor activity	MF	21/152	1.20 × 10^−5^	GPR34/ADGRE3/PIGR/TLR4/CCR8/ANTXR2/PTAFR/IFNAR2/CX3CR1/FZD4/IL5RA/C5AR2/FAS/—/PLXNB2/GPBAR1/CTSH/GPR84/ABCA1/C5AR1
Serine-type endopeptidase activity	MF	7/152	0.000321896	TMPRSS2/HP/CTSH/C1RL/CFB
Serine-type peptidase activity	MF	7/152	0.000858309	TMPRSS2/HP/CTSH/C1RL/CFB
Serine hydrolase activity	MF	7/152	0.00118412	TMPRSS2/HP/CTSH/C1RL/CFB
Amino acid transmembrane transporter activity	MF	5/152	0.001412487	SLC38A1/MGC157082
External side of plasma membrane	CC	8/167	0.00311	TLR4/CCR8/ANTXR2/CX3CR1/MS4A2/IL5RA/ABCA1/ADA
Plasma membrane region	CC	6/167	0.025913	IFIT5/BBS4/TJP1/C5AR2/DLC1/PLEK/CD36/CAMK2N1/DAGLB/TRPM6/C5AR1
Lipid particle	CC	11/167	0.031392	TLR4/CCR8/ANTXR2/CX3CR1/FZD4/MS4A2/IL5RA/FAS/ABCA1/RHOB/ADA

Gene ratio: the ratio of the number of differential genes annotated to the GO number to the total number of differential genes. BP: biological process; MF: molecular function; CC: cellular component.

**Table 3:** KEGG signaling pathway analysis of DEGs.

KEGG signaling pathway	No.	*p*	Genes
JAK–STAT signaling pathway	10	9.67 × 10^−5^	CDKN1A/IL15/IFNAR2/IL5RA/IL13RA1/IRF9/CCND3/PIAS3/CREBBP/PIAS1
Influenza A	11	1.20 × 10^−4^	TLR4/HSPA2/TMPRSS2/NFKBIA/IFNAR2/IRF9/FAS/BOLA-DQB/CREBBP
Asthma	4	2.12 × 10^−3^	FCER1A/MS4A2/BOLA-DQB
Cytokine–cytokine receptor interaction	11	2.62 × 10^−3^	IL15/CCR8/IFNAR2/CX3CR1/IL1RN/IL5RA/CCR6/IL13RA1/CCL24/FAS/
TNF signaling pathway	4	4.88 × 10^−3^	IL15/NFKBIA/FAS/CASP3
Fructose and mannose metabolism	3	5.48 × 10^−3^	PFKFB3/FBP1/SORD
Nitrogen metabolism	4	7.40 × 10^−3^	CA8/GLUL/GLUD1/CPS1
IL-17 signaling pathway	4	8.64 × 10^−3^	NFKBIA/CASP3/USP25/RELA
Toll-like receptor signaling pathway	5	8.68 × 10^−3^	TLR4/LY96/NFKBIA/IFNAR2/
NOD-like receptor signaling pathway	7	1.34 × 10^−2^	TXNIP/TLR4/ANTXR2/NFKBIA/IFNAR2/IRF9/CASP4
NF-kappa B signaling pathway	5	1.34 × 10^−2^	TLR4/LY96/NFKBIA/TNF-*α*

## Data Availability

The RNAseq data are available at NCBI GenBank database under accession number PRJNA702464.
